# Supplementary figures and images for: Overgrazing-induced legacy effects may permit Leymus chinensis to cope with herbivory
Source: PeerJ. 2020 Oct 8;8:e10116. doi: 10.7717/peerj.10116 (PMC7548072; doi:10.7717/peerj.10116)

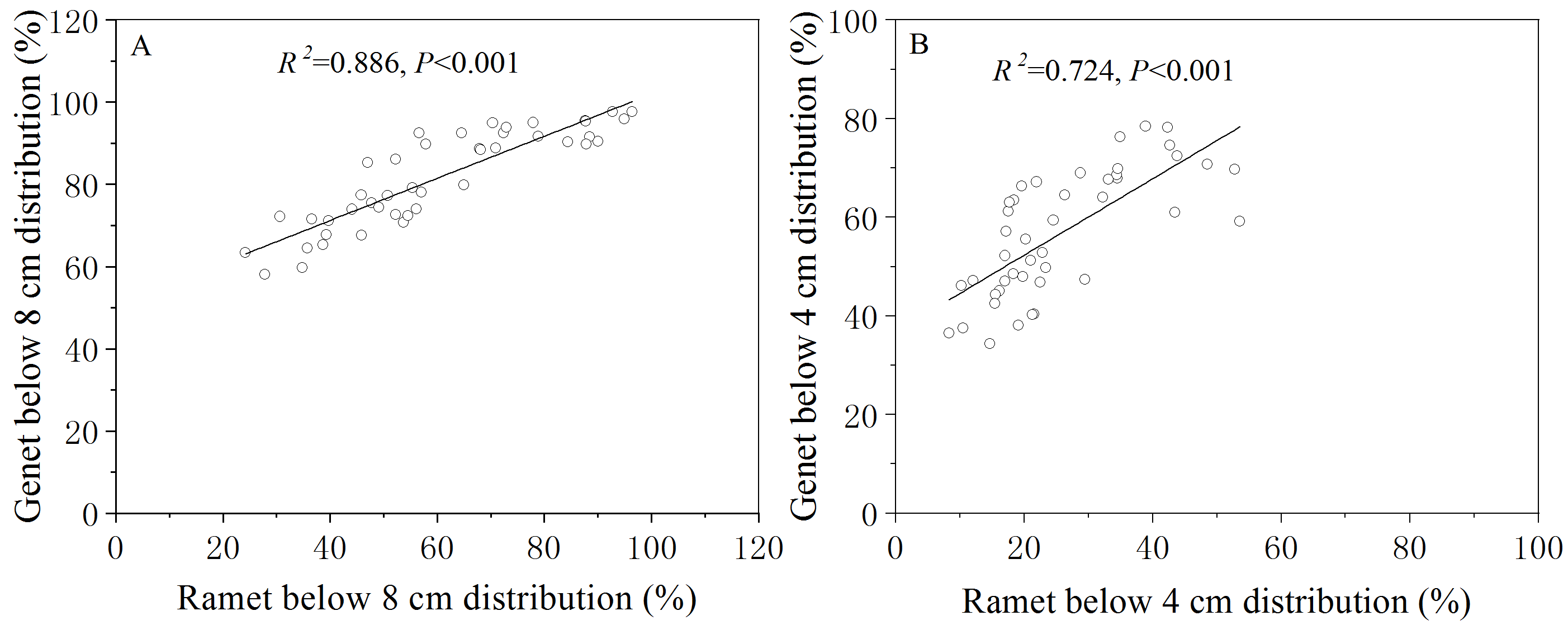

Supplement: Supplemental Information 1 — (A) Below eight cm distribution; (B) below four cm distribution. [file peerj-08-10116-s001.png]

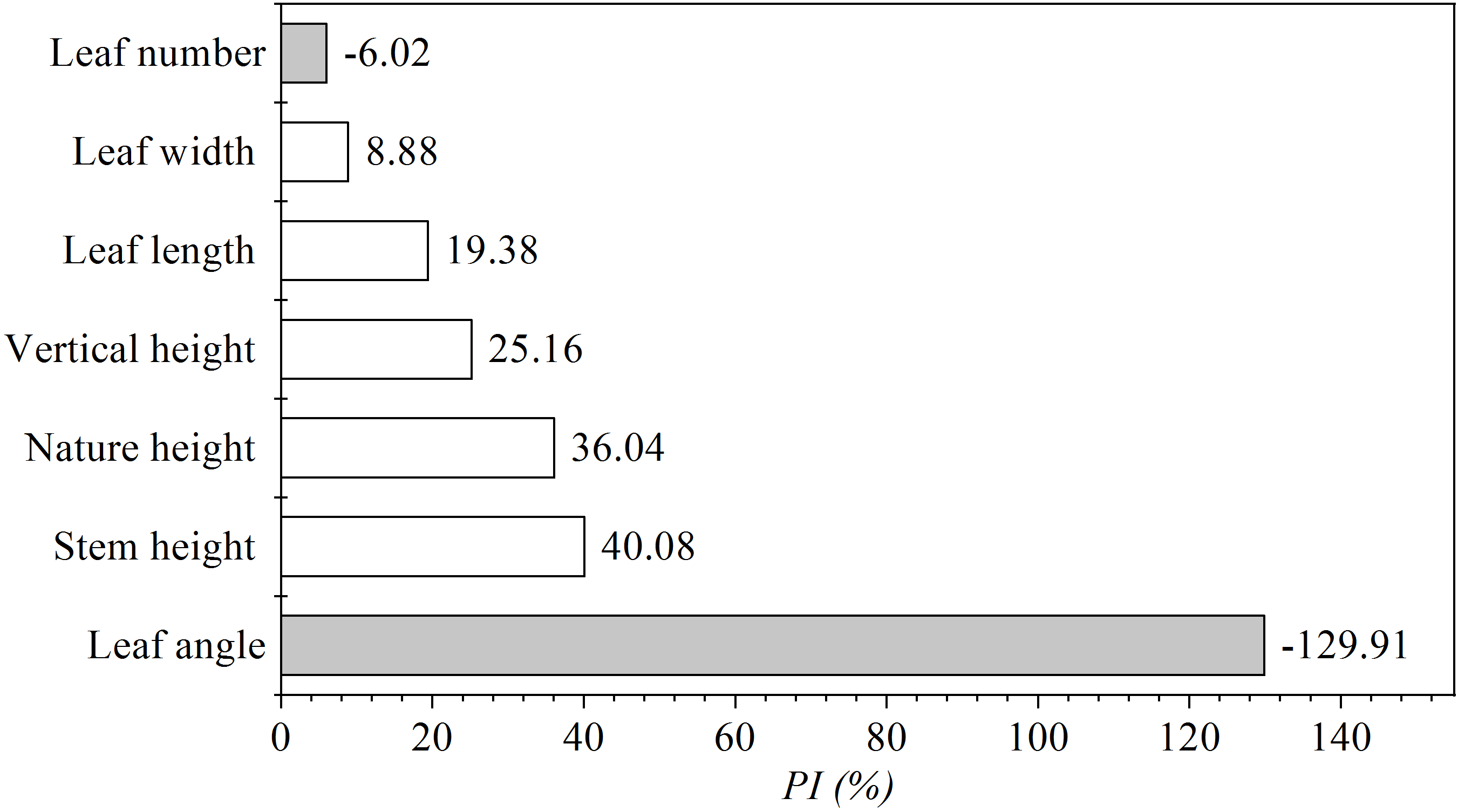

Supplement: Supplemental Information 2 — The light grey bars (i.e., Leaf angle and Leaf number) indicate that the trait of OG is larger than NG. [file peerj-08-10116-s002.png]

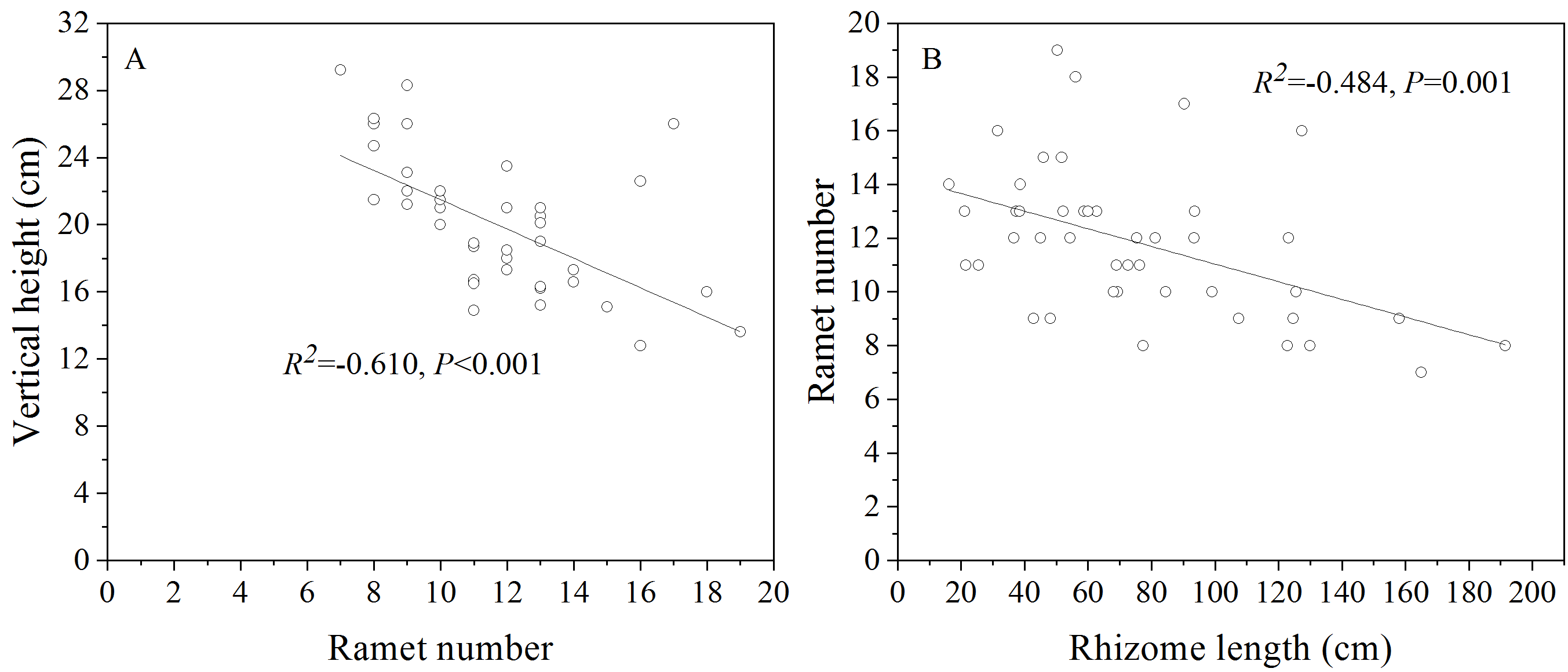

Supplement: Supplemental Information 3 [file peerj-08-10116-s003.png]

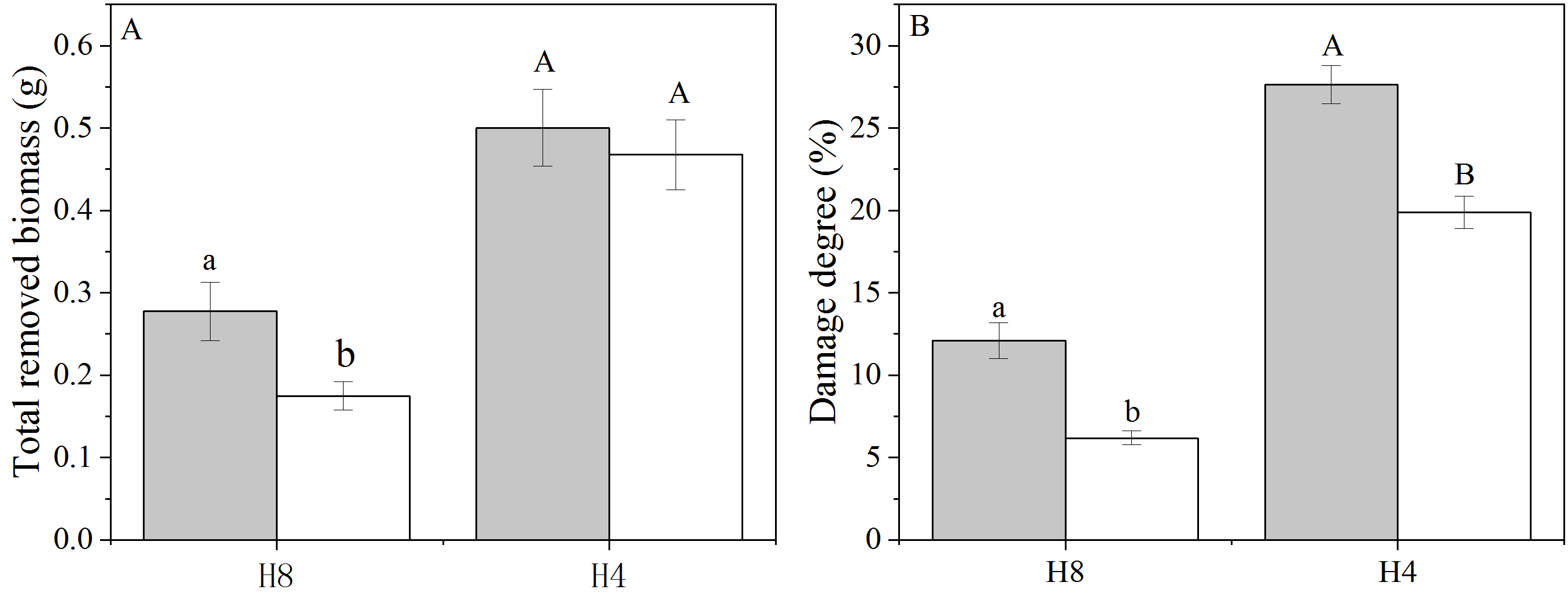

Supplement: Supplemental Information 4 — H8: Simulated moderate grazing; H4: Simulated heavy grazing. The grey bars indicate L. chinensis genets collected from the grazing exclusion plot (NG) while the white bars indicate L. chinensis genets collected from the continuously grazed plot (OG). The damage degree was calculated with “(total removed biomass)/total biomass”. The different lowercase letters indicate the significant differences of total removed biomass and damage degree between NG and OG under H8 simulated grazing treatment while the different capital letters indicate the significant differences between NG and OG under H4 simulated grazing treatment. [file peerj-08-10116-s004.png]
