# Supplementary material for: Overgrazing-induced legacy effects may permit Leymus chinensis to cope with herbivory
Source: PeerJ. 2020 Oct 8;8:e10116. doi: 10.7717/peerj.10116 (PMC7548072; doi:10.7717/peerj.10116)
Supplement: Supplemental Information 5 [file peerj-08-10116-s005.docx]

|  | MS | SH | LL | LW | VH | LA | B4B | A4B |
| --- | --- | --- | --- | --- | --- | --- | --- | --- |
| SH | 0.63 | 0 | 0 | 0 | 0 | 0 | 0 | 0 |
| LL | 0.753 | 0 | 0 | 0 | 0 | 0 | 0 | 0 |
| LW | 0.307 | 0 | 0 | 0 | 0 | 0 | 0 | 0 |
| VH | 0.739 | 0.48 | 0.58 | 0 | 0 | 0 | 0 | 0 |
| LA | -0.973 | 0 | 0 | 0 | 0 | 0 | 0 | 0 |
| B4B | -0.59 | 0 | 0 | 0 | 0 | 0.606 | 0 | 0 |
| A4B | 0.608 | 0.339 | 0.41 | 0.279 | 0.707 | 0 | 0 | 0 |
| B4D | -0.731 | -0.208 | -0.251 | -0.171 | -0.433 | 0.369 | 0.609 | -0.612 |

^[[1]](#endnote-1)^ MS: materials source; SH: stem height; LL: Leaf Length; LW: Leaf width; VH: vertical height; LA: leaf angle; B4B: the ramet above-ground biomass accumulation below 4 cm; A4B: the ramet above-ground biomass accumulation above 4 cm; B4D: The vertical distribution of above-ground biomass below 4 cm.

1. [↑](#endnote-ref-1)
