# Supplementary material for: Overgrazing-induced legacy effects may permit Leymus chinensis to cope with herbivory
Source: PeerJ. 2020 Oct 8;8:e10116. doi: 10.7717/peerj.10116 (PMC7548072; doi:10.7717/peerj.10116)
Supplement: Supplemental Information 6 [file peerj-08-10116-s006.docx]

|  | MS | LL | SH | LA | LW | NH | B8B | A8B |
| --- | --- | --- | --- | --- | --- | --- | --- | --- |
| LL | 0.668 | 0 | 0 | 0 | 0 | 0 | 0 | 0 |
| SH | 0.668 | 0 | 0 | 0 | 0 | 0 | 0 | 0 |
| LA | -0.972 | 0 | 0 | 0 | 0 | 0 | 0 | 0 |
| LW | 0.328 | 0 | 0 | 0 | 0 | 0 | 0 | 0 |
| NH | 0.826 | 0.319 | 0.429 | 0 | 0 | 0 | 0 | 0 |
| B8B | -0.495 | 0 | 0 | 0.509 | 0 | 0 | 0 | 0 |
| A8B | 0.698 | 0.24 | 0.323 | 0 | 0.234 | 0.753 | 0 | 0 |
| B8D | -0.766 | -0.205 | -0.275 | 0.175 | -0.199 | -0.642 | 0.344 | -0.853 |

^[[1]](#endnote-1)^ B8B: the ramet above-ground biomass accumulation below 8 cm; A8B: the ramet above-ground biomass accumulation above 8 cm; B8D: The vertical distribution of above-ground biomass below 8 cm; LA: leaf angle; NH: natural height; Other abbreviations were the same meaning as Table S1.

1. [↑](#endnote-ref-1)
